# Differential progression of unhealthy diet-induced hepatocellular carcinoma in obese and non-obese mice

**DOI:** 10.1371/journal.pone.0272623

**Published:** 2022-08-22

**Authors:** Emma Hymel, Elizabeth Vlock, Kurt W. Fisher, Paraskevi A. Farazi

**Affiliations:** 1 Department of Epidemiology, College of Public Health, University of Nebraska Medical Center, Omaha, NE, United States of America; 2 Nebraska Center for the Prevention of Obesity Diseases through Dietary Molecules, College of Education and Human Sciences, University of Nebraska Lincoln, Lincoln, NE, United States of America; 3 Department of Pathology and Microbiology, College of Medicine, University of Nebraska Medical Center, Omaha, NE, United States of America; Universidade do Estado do Rio de Janeiro, BRAZIL

## Abstract

**Background:**

Non-alcoholic fatty liver disease (NAFLD) ranks first among liver diseases in Western countries. NAFLD is typically associated with obesity and diabetes, however it also develops in lean individuals without metabolic syndrome. The prevalence of lean NAFLD is 7 percent in the U.S. and 25–30 percent in some Asian countries. NAFLD starts with excess liver fat accumulation (NAFL), progresses to nonalcoholic steatohepatitis (NASH), cirrhosis and hepatocellular carcinoma (HCC). The pathogenesis of lean NASH-HCC and how it differs from obese NASH-HCC is not well understood.

**Methods:**

In this work, we generated a mouse model of lean and obese NASH-HCC using a choline deficient/high trans-fat/fructose/cholesterol diet and a choline supplemented/high trans-fat/fructose/cholesterol diet, respectively, to compare progression to NASH-HCC in lean versus obese mice. Comparisons were made at the organismal, histological, and molecular level by investigating fatty acid metabolism in the plasma of these mice.

**Results:**

Obese mice showed more pronounced glucose intolerance and insulin resistance, higher levels of plasma cholesterol and triglycerides, and higher penetrance of NASH compared to lean mice. Despite the abnormal metabolic profile of obese mice, male obese and lean mice developed HCC with similar penetrance (53.3% and 53.8%, respectively), albeit lean mice showed faster tumor progression as evidenced by the larger tumor size and lower HCC-free survival. None of the female lean mice developed HCC, while 50% of female obese mice developed HCC. Both groups of mice showed a reduction in plasma polyunsaturated fatty acids (PUFAs), however, the levels were higher towards the endpoint in obese mice compared to lean mice.

**Conclusions:**

Unhealthy diet composition appears to drive progression to NASH-HCC rather than the organismal effects of obesity. PUFA levels may increase due to systemic inflammation in obese mice and act as suppressors of tumor progression, thus delaying HCC progression in obese mice compared to lean mice. These models could be used to further dissect the molecular pathogenesis of lean and obese NASH-HCC and address the mechanisms whereby PUFAs may be implicated in hepatocarcinogenesis.

## Background

Globally, the prevalence of non-alcoholic fatty liver disease (NAFLD) is 24 percent and is predicted as the leading cause for liver transplantation in the United States by 2030 [1,2]. NAFLD encompasses a liver disease spectrum that starts with excess accumulation of fat in the liver (NAFL) and progresses to nonalcoholic steatohepatitis (NASH) with inflammation, cirrhosis and finally hepatocellular carcinoma (HCC) [3]. An increasing number of HCCs are caused by NAFLD [4–6].

Some of the risk factors for NAFLD include obesity, diabetes, and genetic predisposition, however, it can also develop in lean individuals that do not have metabolic syndrome. The prevalence of lean NAFLD in the U.S. is 7 percent and as high as 25–30 percent in certain Asian countries [2]. Genetic mutations resulting in triglyceride accumulation in the liver contribute to NAFLD in some patients [7], but more typically diet-related visceral obesity (diet high in fructose, fat, and cholesterol) is associated with lean NAFLD. Patients with lean NAFLD do not exhibit all the co-morbidities of metabolic diseases, such as insulin resistance, higher serum cholesterol and triglyceride levels, or higher liver enzymes that obese patients exhibit. Since risk factors for lean NAFLD are still not well understood, identification of lean NAFLD is difficult and results in delayed diagnosis and poor prognosis [8,9].

NAFLD progression is characterized by lipogenesis and lipid metabolism changes, such as alterations in serum fatty acids of NASH patients [10,11]. Currently lipid metabolism changes during progression to HCC are not well understood. Certain phospholipids and ceramides show lower levels in human HCC tissues compared to normal tissues [12]. Furthermore, a genetic murine model of NASH revealed distinct patterns of serum and tissue fatty acid levels that correlate with early-stage HCC [13]. Higher levels of free fatty acids were observed in the livers of mice with diet-induced NAFLD [14] and polyunsaturated fatty acids were reduced in the plasma of mice with lean NASH-HCC [15].

Various mouse models of diet-induced HCC have been generated, however, they are characterized by certain limitations: 1) they develop diet-induced HCC in the context of obesity and/or 2) they develop HCCs with low penetrance, or 3) they develop HCCs with high penetrance after a very long duration of feeding (80 weeks) [16–19]. A model of lean NASH-HCC was recently developed by utilizing a choline deficient, high trans-fat/sucrose/cholesterol diet to induce NASH-HCC in mice of the C57BL/6N strain [15]. In this work we refined the aforementioned mouse model by adding fructose to the diet instead of sucrose, since fructose is directly converted to fat in the liver. In addition, we used the same high fat diet formulation in the context of choline supplementation, in order to generate an obese model of NASH-HCC. The aim was to develop an obese and non-obese (lean) mouse model of NASH-HCC, in which the only difference in the diet composition would be the presence and absence of choline, respectively. These mouse models would enable further molecular comparisons of lean and obese NASH-HCC.

## Materials and methods

### Animals and experimental diets

The Institutional Animal Care and Use Committee at the University of Nebraska Medical Center approved this study (Protocol #: 17–018) and it was also conducted in compliance with the ARRIVE guidelines. Male (n = 103) and female (n = 25) C57BL/6N mice (Charles River Laboratories) were allowed to acclimate and housed as previously described starting at 3 weeks of age [15]. They were housed in a temperature-, humidity-, and ventilation-controlled vivarium on a 12-h light/dark cycle in specific pathogen-free conditions.

30 males and 10 females were fed with a choline supplemented, high trans-fat, fructose, and cholesterol diet (CS-HFFC; D18091706), 38 males and 10 females were fed with a choline deficient, high trans-fat, fructose, and cholesterol diet (CD-HFFC; D17071001), and 35 males and 5 females were fed with a low-fat control diet (CON; D16120211; Research Diets, New Brunswick, New Jersey; S1 Table). Choline deficiency results in lean NASH-HCC [15], whereas choline supplementation allows for liver carcinogenesis in the context of obesity [20]. The sample size was estimated based on HCC penetrance in our previous work [15]. Food consumption was monitored, mice were weighed and regular husbandry checks were performed as previously described [15].

### Glucose and insulin tolerance test

Intraperitoneal glucose tolerance tests (IPGTT) and intraperitoneal insulin tolerance tests (IPITT) were performed every 12 weeks starting at 12 weeks of age. Glucose (2g/kg) or insulin (0.5 units/kg) was administered through intraperitoneal injection after mice were fasted for 6 hours in the morning. Blood samples were obtained from the tail vein and blood glucose levels were measured as previously described [15] before glucose or insulin challenge (0 minutes) as well as at 15-, 30-, 60-, and 120-minutes post injection. An additional value was taken at 45 minutes after insulin injection. Blood glucose levels were graphed as previously described [15,21]. Individual baseline blood glucose measurements before glucose administration were used as reference (t = 0).

### Measurement of plasma lipids and liver enzymes

Every 12 weeks beginning at 12 weeks of age whole blood was collected from all mice to assess plasma lipids (cholesterol, triglycerides) and liver enzymes (alanine aminotransferase, ALT; aspartate aminotransferase, AST) as previously described [15].

### Liver biopsy

Liver biopsies were performed on all mice at 20 weeks of age to assess liver disease after 16 weeks on specialized diets. All animals were weighed and received preoperative IP injections of carprophen (5mg/kg). Anesthesia was induced with 3–5% isoflurane and maintained with 1–2% isoflurane during surgery. Anesthesia depth was verified every 10 minutes using a pedal pinch and recorded appropriately. Mice were placed in dorsal recumbency and prepared aseptically for surgery. 50-100mg of tissue was biopsied from the right lobe and absorbable gelatin sponge was used to stop any bleeding. At the completion of surgery, each animal was observed until mice regained sternal recumbency and then were returned to their home cage. Mice were observed daily and received additional analgesic injections for two days postoperatively. Skin staples were removed after 10–14 days. Half of the biopsied tissue was frozen, and the other half was fixed in 10% formalin to be paraffin embedded.

### Histological evaluation

Mice were monitored until the endpoint of the study (64 weeks of age) and were euthanized per institutional ethical guidelines by CO_2_ inhalation if they showed signs of poor health. Tissues were harvested for analysis. Exsanguination was used to confirm death. A cardiac puncture was performed to collect blood right after euthanasia. Postmortem, livers were excised, weighed, and observed grossly for the appearance of nodules. Tissue samples were snap frozen in liquid nitrogen and stored at -80°C and the remaining tissues were fixed in 10% formalin for 2 hours and paraffin embedded at the Tissue Sciences Facilities at the University of Nebraska Medical Center. Each nodule observed grossly was cut from the rest of the liver and put in an individual cassette for histological assessment. The rest of the liver that had no visible nodules was cut into smaller 2-3mm pieces in thickness which were laid out horizontally in cassettes to provide a cross-sectional view of the entire liver and allow for counting of microscopic nodules not visible by naked eye. This allowed an accurate quantification of macroscopic and microscopic lesions across the different diet groups. Tissue sections were stained with Hematoxylin and Eosin (H&E) and Masson-Trichrome. At necropsy, an additional reticulin stain was performed. These stained sections were scored blindly by an experienced pathologist. Score were recorded for steatosis, ballooning, and inflammation in order to determine the presence of non-alcoholic fatty liver disease (NAFLD) and non-alcoholic steatohepatitis (NASH) [22]. Steatosis was scored as follows: 0 = <5%, 1 = 5–35%, 2 = 36–64%, and 3 = >65% cells had macrovesicular steatosis. Ballooning was scored as follows: 1 = rare ballooned degeneration or apoptotic cells, 2 = clusters of ballooned hepatocytes with apoptotic cells, 3 = clusters of ballooned hepatocytes with apoptotic cells and clusters of foamy macrophages. Inflammation was scored as follows: 1 = 1 focus/High Power Field (HPF), 2 = >1 focus/HPF, 3 = diffuse inflammation. Fibrosis was scored as follows: 1 = pericentral, 2 = pericentral and periportal, 3 = bridging, 4 = cirrhosis. The stained liver sections were also evaluated for the presence of regenerative nodules, dysplastic nodules, and hepatocellular carcinomas as previously described [15].

### Metabolomics

Plasma collected at 24, 48, and 64 weeks of age and tissue collected at 64 weeks of age were analyzed for fatty acid levels. Plasma and tissue samples were randomly selected from mice fed with the CD-HFFC diet that had HCC (*n* = 6), mice fed with the CS-HFFC diet that had HCC (n = 6), and mice fed with the control diet (n = 6). Mice from both CD-HFFC (n = 3) and CS-HFFC (n = 3) diets that only developed dysplastic nodules were only analyzed at 64 weeks of age (plasma and tissue). All mice selected were males.

Plasma (*n* = 60) and tissue (*n* = 24) samples were prepared for analysis as previously described [15] and extracts were sent to the Lipidomics Core Facility at Wayne State University for fatty acid metabolomics analysis as previously described [15]. In total, 32 fatty acids (C12-C26) were analyzed (S2 Table).

### Cytokine analysis

Plasma collected from 32 weeks of age were analyzed for cytokine levels using the R&D Systems Proteome Profiler Mouse Cytokine Array Kit, Panel A (ARY006; Minneapolis, MN). Plasma samples were randomly selected from mice that developed HCC from the CD-HFFC (n = 3) and the CS-HFFC diet (n = 3). All mice selected were males.

Briefly, samples were incubated on membranes containing antibodies for 40 cytokines in duplicate (S3 Table). After an overnight incubation at 4°C, membranes were washed and incubated with a streptavidin secondary antibody. A chemiluminescent solution was applied, and membranes were exposed to film for 1–10 minutes.

Resulting films were scanned and spot intensity was calculated using Adobe Photoshop. Each cytokine signal was measured using the lasso tool and the mean and pixel density was recorded and averaged. The averaged mean and pixel density values were multiplied to get the absolute intensity value for each cytokine. Data were expressed in relative intensity (absolute intensity of cytokine/relative intensity of control) and graphed.

### Statistical analyses

SAS 9.2 software (SAS Institute Inc, Cary, North Carolina) was used for data analysis. Continuous data were expressed as mean +/- standard deviation or as absolute number or percentage for categorical variables. Diet-based group comparisons were performed using a one-way ANOVA. Survival curves were generated using GraphPad Prism 6 software. Survival was compared between mice fed with the CD-HFFC diet and mice fed with the CS-HFFC diet that died before the endpoint due to any health-related cause or due to HCC. Mice that were euthanized at the endpoint were censored irrespective of whether they developed HCC since they were otherwise healthy and therefore, we could not designate the cause of death as HCC. A *P*- value < 0.05 was considered statistically significant for all comparisons.

## Results

### Assessment of weight gain

**Fig 1:**
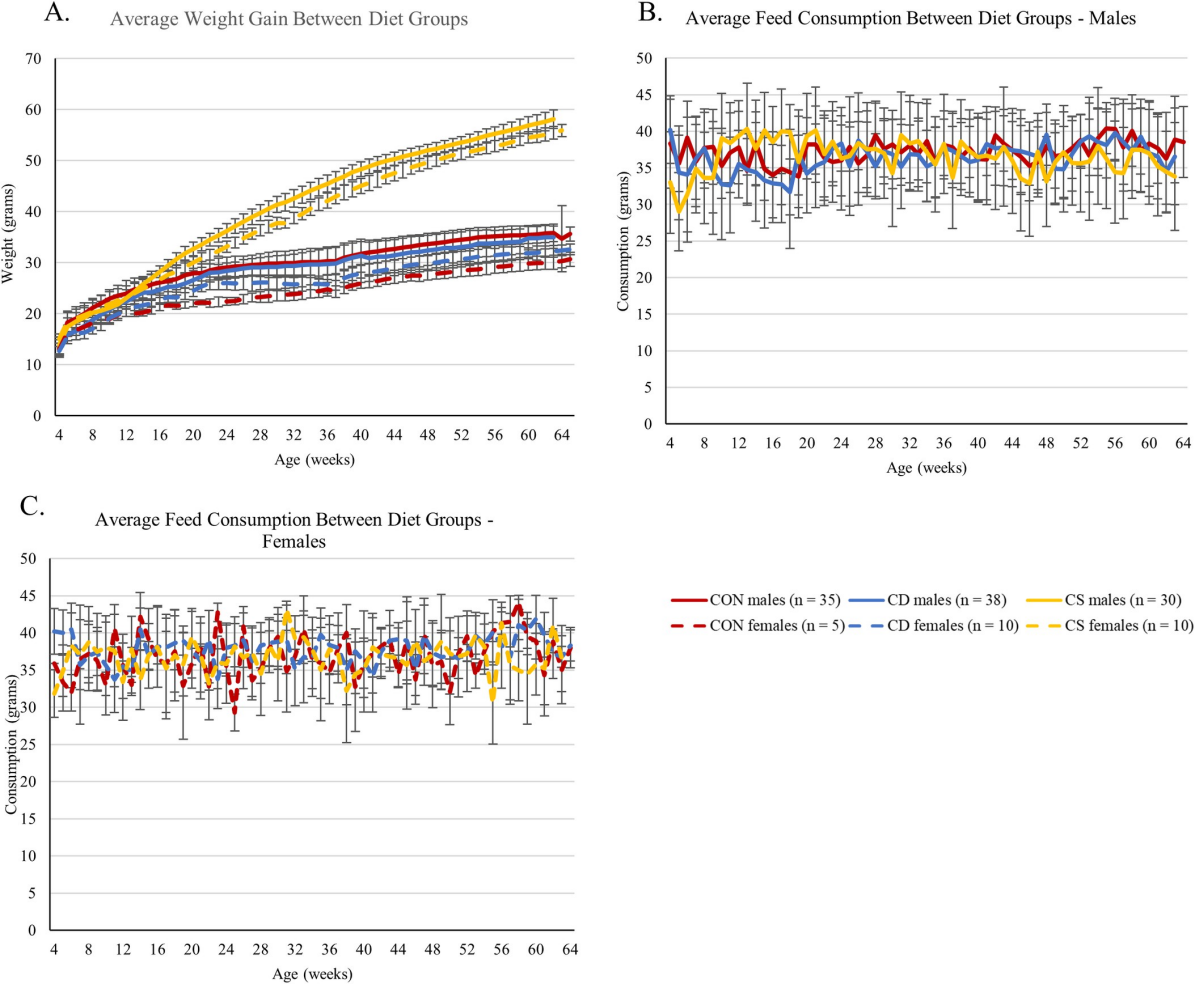
Weight gain and food consumption. (A) Weight gain of mice fed with the control, CD-HFFC, and CS-HFFC diets. Male and female mice fed with the CS-HFFC diet gained significantly more weight than mice fed with the control or CD-HFFC diets. Male CS-HFFC fed with mice were considered obese at 14 weeks of age while females fed with the same diet became obese at 10 weeks of age. (B) and (C) Feed consumption assessment per cage. Overall, the three groups of mice seem to be consuming the same amount of feed.

Male mice fed with the CS-HFFC diet weighed significantly more than mice on either the CD-HFFC or control diet by 14 weeks of age (p = 0.036; Fig 1A). By 64 weeks of age these mice weighed 66% and 62% more than mice on the CD- HFFC and control diet, respectively. Similarly, the female mice on the CS-HFFC diet became obese by 10 weeks of age (p = 0.026; Fig 1A) with a total weight difference of 72% and 84% compared to CD- HFSC and the control diet, respectively. Furthermore, CD-HFFC and control male mice gained an average of 154% and 171% of their starting weight respectively, while male CS-HFFC mice gained an average of 319%. Female CD-HFFC and control mice gained 154% and 131% of their original body weight respectively, whereas CS-HFFC females gained 266% of their original body weight (S4 Table). All mice were consuming the same amount of food on average (p = 0.07 and 0.814 for males and females, respectively, Fig 1B), which was measured crudely by weighing the amount of feed left in the cage before changing feed.

### Survival

**Fig 2:**
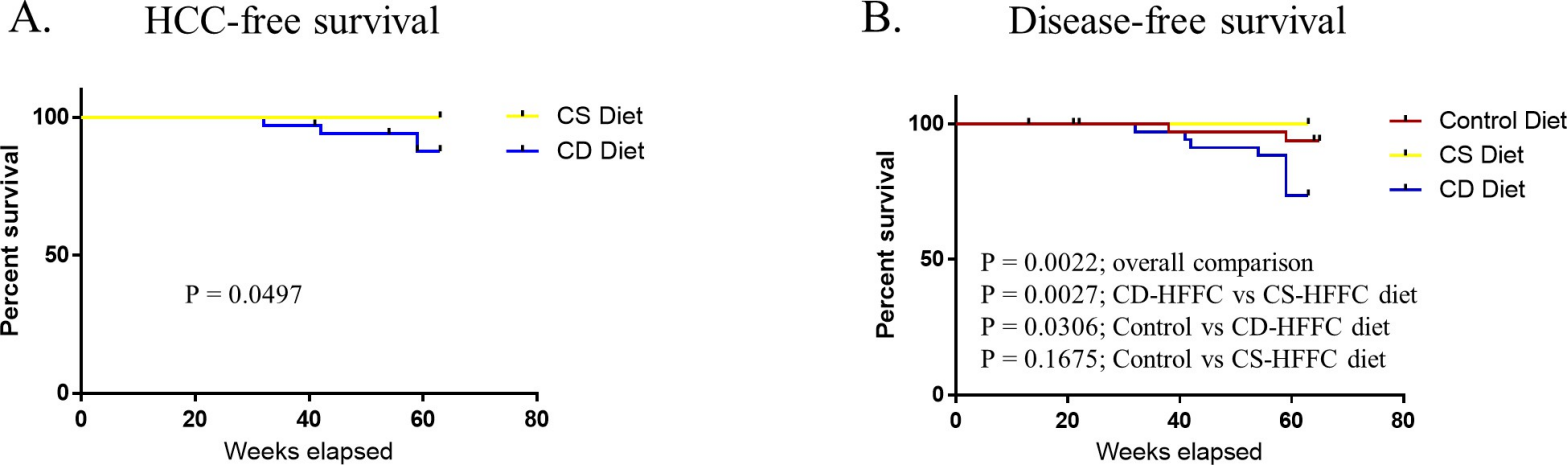
Survival curves. (A) HCC-free, and (B) Disease-free survival of male mice fed with the different diet types. Mice fed with the CD-HFFC diet had poorer disease-and HCC-related survival.

Mice fed with the CS-HFFC diet survived until the endpoint. In contrast, mice fed with the CD-HFFC had a lower overall health problem-free survival rate compared to mice fed with the CS-HFFC and control diet. Furthermore, mice fed with the CD-HFFC diet had a lower HCC-free survival rate compared to mice fed with the CS-HFFC diet (Fig 2).

### Assessment of glucose tolerance and insulin resistance

**Fig 3:**
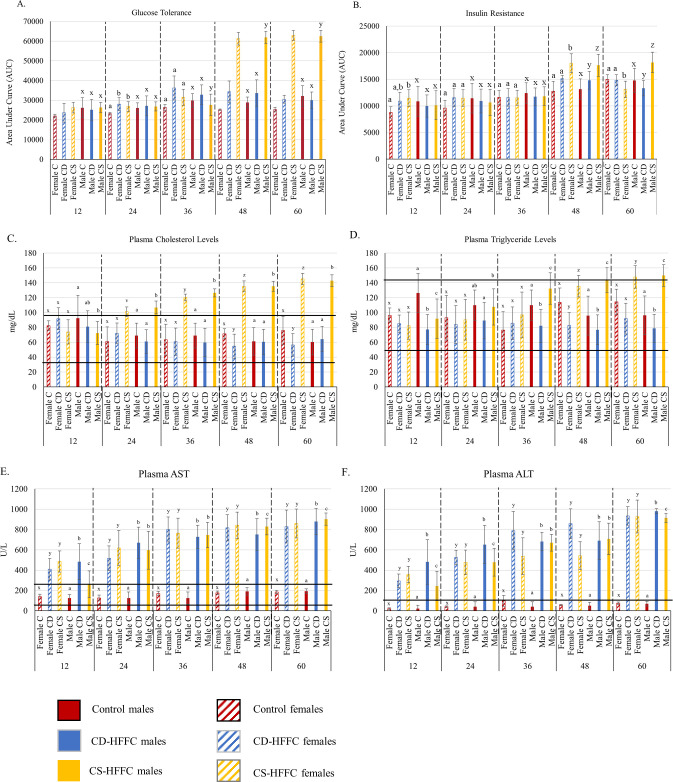
Metabolic function, plasma lipids, and plasma enzymes in mice fed with the control (C), CD-HFFC (CD), and CS-HFFC (CS) diets. (A) Glucose tolerance. Both male and female mice fed with the CS-HFFC diet exhibited intolerance to glucose at 48 (p = 1.11 x 10–16, 1.02 x 10–13) and 60 (p = 1.11 x 10–16, 1.11 x 10–16) weeks of age. (B) Insulin resistance. No differences were observed until 48 weeks of age. Male and female mice fed with the CS-HFFC diet were insulin resistant at both 48 (p = 5.41 x 10–13, 5.31 x 10–6) and 60 (p = 7.59 x 10–13, 0.015) weeks of age. (C) Plasma cholesterol and (D) plasma triglyceride levels. (E) Plasma AST and (F) plasma ALT levels. Horizontal black lines indicate lower and upper limits of normal plasma lipid and enzyme levels. Statistical differences between diet groups are shown with either a,b,c or x,y,z, markings above the graphs (one set used for males and the other for females), where statistically significant differences would be denoted by different letters across diets.

Male mice fed with the CS-HFSC diet developed glucose intolerance and insulin resistance by 48 weeks (p = 1.11 x 10^−16^ and 5.41 x 10^−13^) and remained in this state at 60 weeks (p = 1.11 x 10^−16^ and 7.59 x 10^−13^) weeks of age. Female mice fed with the CS-HFFC diet developed insulin resistance transiently at around 48 weeks and were glucose intolerant starting at 48 weeks until the endpoint. In contrast, only female CD-HFFC fed with mice developed glucose intolerance after 24 weeks and only males developed insulin resistance transiently at around 48 weeks (Fig 3A and 3B).

### Assessment of plasma lipid profile and liver damage

CS-HFFC fed males and females had significantly higher levels of cholesterol after 24 weeks of age while there were no differences between CD-HFFC fed and control diet fed mice. However, male CD-HFFC fed mice had lower plasma cholesterol compared to CS-HFFC fed mice after 48 weeks. Male CD-HFFC mice had lower levels of triglycerides compared to control and CS-HFSC mice after 12 weeks (Fig 3C and 3D), albeit all groups had triglyceride levels within the normal range. Both AST and ALT liver enzymes were significantly higher in both male and female mice fed with the CS-HFSC and CD-HFSC diet compared to control diet fed mice. Male CD-HFFC fed mice showed higher plasma liver enzymes at 12 (AST; p = 8.58 × 10^−10^ and ALT; p = 1.11 × 10^−16^) and 24 weeks (ALT; p = 1.11 × 10^−16^) compared to CS-HFFC fed mice (Fig 3E and 3F). There were no differences in plasma AST or ALT levels among females fed with the CD-HFFC and CS-HFFC diet.

### Assessment of liver disease at midpoint

**Fig 4:**
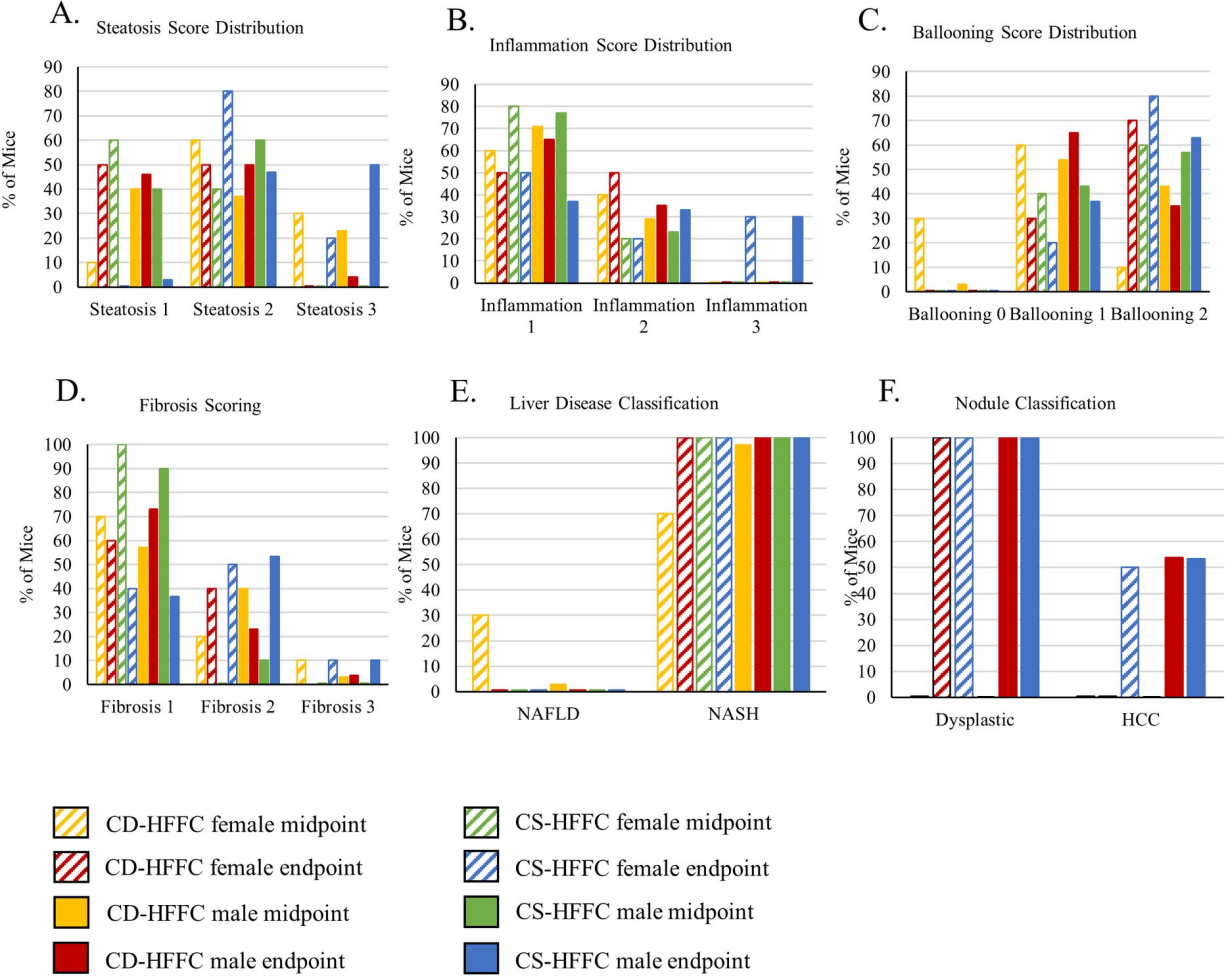
Descriptive results from liver biopsy at study midpoint and necropsy at study endpoint. (A) Distribution of steatosis, (B) inflammation, and (C) ballooning scores at study midpoint and endpoint. (D) Fibrosis scoring at midpoint and endpoint. Both male and female mice fed with the CD-HFFC diet developed a higher degree of fibrosis than mice fed with the CS-HFFC diet at the study midpoint, however CS-HFFC fed mice develop a higher degree of fibrosis by the study endpoint. All mice fed with the control diet had a fibrosis score of 0 and had normal liver phenotypes. (E) Liver disease diagnosis. All mice fed with the CS-HFFC diet developed NASH by study midpoint. All mice fed with both the CD-HFFC and CS-HFFC diet developed NASH by study endpoint. (F) Prevalence of HCC at study endpoint (64 weeks of age).

**Fig 5:**
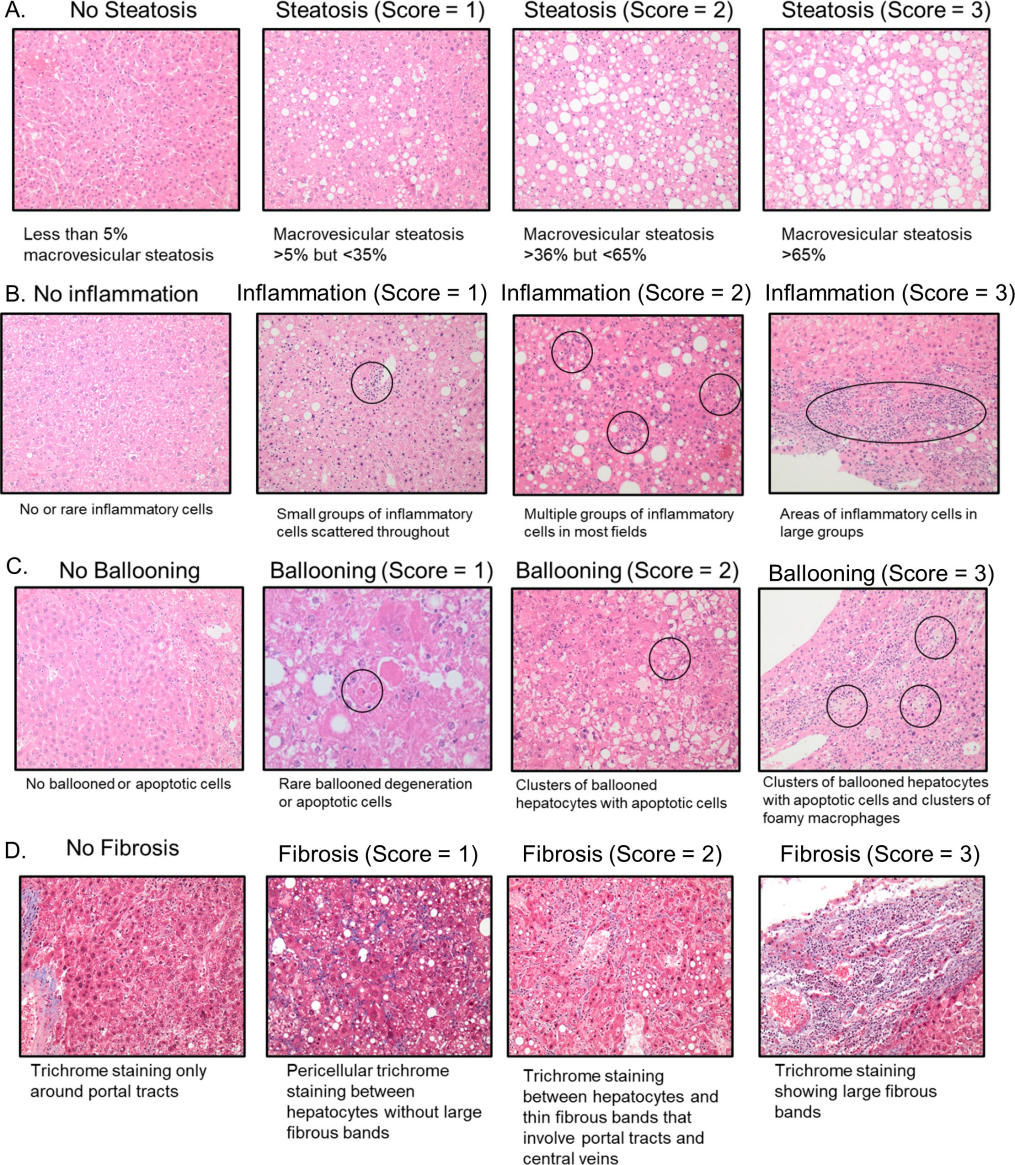
Histological pictures showing the various degrees of steatosis, inflammation, ballooning and fibrosis. (A) Steatosis scores of 1–3. (B) Inflammation scores of 1–3. (C) Ballooning scores of 1–3. (D) Fibrosis scores of 1–3.

We assessed liver steatosis, inflammation, and ballooning at 20 weeks of age. 8 male (23%) and 3 female (30%) CD-HFFC mice had steatosis scores of 3, whereas none of the CS-HFFC fed mice had steatosis scores of 3 at that timepoint (Fig 4A; S9 Table). While there were no significant differences observed in the average steatosis scores between CD-HFFC and CS-HFFC fed males, 60% of the females fed with the CS-HFFC diet received a steatosis score of 1, bringing the average score down enough for it to be significantly lower than that of the CD-HFFC females (1.4 vs 2.2, respectively; p = 0.006; S8 Table). Males fed with both the CD-HFFC and CS-HFFC diet had similar degree of liver inflammation, with 25 (71%) and 23 (77%) scoring a 1. Similarly, in the females, the majority of both CD-HFFC (60%) and CS-HFFC (80%) mice had a liver inflammation score of 1 (Fig 4B; S9 Table). The average inflammation score was not different for the males, however there were significant differences in the scores of the CD-HFFC and CS-HFFC females (1.4 vs 1.2, respectively; p = 0.006; S8 Table). Finally, there were no observed differences in the hepatocyte ballooning scores for either males or females fed with the CD-HFFC and CS-HFFC diets (p = 0.0683 males; 0.355 females; S8 Table). 17 (57%) of the males fed with the CS-HFFC diet had a ballooning score of 2 (on a 0–2 scale) while only 15 (43%) of the CD-HFFC male mice received the same score (Fig 4C; S9 Table). Representative histological pictures of steatosis, inflammation, ballooning, and fibrosis are presented in Fig 5 (Fig 5).

By 20 weeks of age, 30 (100%) and 34 (97.2%) male mice fed with CS-HFFC and CD-HFFC diets developed NASH, respectively while 10 (100%) and 7 (70%) female mice fed with CS-HFFC and CD-HFFC diets developed NASH, respectively. None of the mice fed with the control diet developed NASH (Fig 4E).

### Assessment of NASH and HCC development at the endpoint

Mice fed with the CD-HFFC and CS-HFFC diets had enlarged livers, significantly higher liver to body weight ratios, and enlarged spleens compared to mice fed with the control diet (S5 and S6 Tables). Male mice fed with the CD-HFFC diet developed significantly larger HCC tumors than CS-HFFC fed males (91.29mm^2^ and 11.25mm^2^, respectively; p = 1.09 x 10^−5^) at the endpoint (S5 and S6 Tables). There were no differences in tumor multiplicity between the two groups.

We also assessed liver disease development. All mice fed with the CD-HFFC and CS-HFFC diets (100%) developed NASH. Neither male (n = 32) nor female (n = 5) mice fed with the control diet developed NASH (Fig 4E). Livers were assessed for degree of steatosis, inflammation, and ballooning. Male mice fed with the CS-HFFC diet had more steatosis at death than their CD-HFFC diet counterparts as 50% (15 mice) of the CS-HFFC mice had a steatosis score of 3 compared to the 4% (1 mouse) of the CD-HFFC fed male mice (Fig 4A; S9 Table). Similarly, 2 (20%) female CS-HFFC mice received a liver steatosis score of 3 whereas none of the CD-HFFC females had a liver steatosis score of 3 (Fig 4A; S9 Table). These numbers were similar for liver inflammation scores as well. Only mice fed with the CS-HFFC diet received an inflammation score of 3 (9 males and 3 females) whereas the majority (65%) of the CD-HFFC fed male mice had an inflammation score of 1 (Fig 4B; S9 Table). Of the 26 male and 10 female CD-HFFC fed mice, 17 (65.4%) males and 3 (30%) females had a ballooning score of 1 and 9 (34.6%) males and 7 (70%) females had a ballooning score of 2, respectively (0–2 scale). For mice fed with the CS-HFFC diet, 11 of the 30 males (36.7%) and 2 of the 10 females (20%) had a ballooning score of 1, and 19 (63.3%) males and 8 (80%) females had a ballooning score of 2 (Fig 4C; S9 Table).

With regards to fibrosis, 19 (73.1%) of the 26 male CD-HFFC fed mice had a fibrosis score of 1, while 6 (23.1%) had a score of 2, and 1 (3.8%) had a score of 3 whereas 11 (36.7%), 16 (53.3%), and 3 (10%) male CS-HFFC fed mice had fibrosis scores of 1, 2 and 3, respectively. For female mice fed with the CD-HFFC diet, 6 (60%) had a fibrosis score of 1 and the remaining 4 (40%) had a fibrosis score of 2. Finally, 4 (40%) females on the CS-HFFC diet had a fibrosis score of 1, while 5 (50%) had a score a 2, and 1(10%) had a score of 3. None of the control mice developed fibrosis (Fig 4D). Representative histological pictures of steatosis, inflammation, ballooning, and fibrosis are presented in Fig 5 (Fig 5).

**Fig 6:**
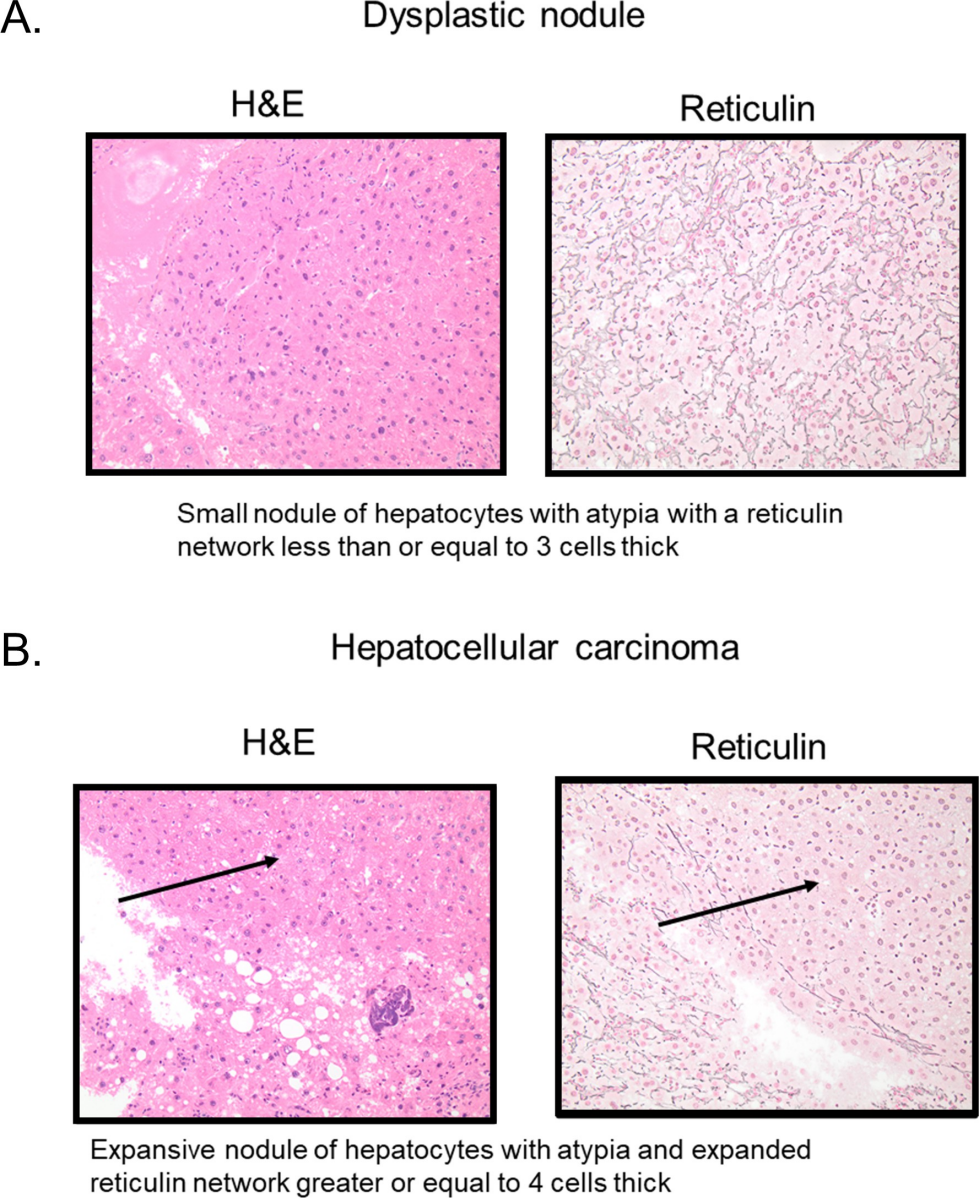
Histological pictures showing representative images of dysplastic nodules and HCC. (A) Micrographs representing dysplastic nodules. (B) Micrographs of hepatocellular carcinoma.

We also assessed the mice for the development of dysplastic nodules and HCCs. Only male mice fed with the CD-HFFC diet developed HCC, with a penetrance of 53.8%. On the contrary, both male and female mice fed with the CS-HFFC diet developed HCC with 53.3% and 50% penetrance, respectively. In addition, all mice fed with both the CD-HFFC and CS-HFFC diet developed dysplastic nodules. None of the control mice developed any type of nodules (Fig 4F). Representative histological pictures of dysplastic nodules and HCC are presented in Fig 6 (Fig 6).

### Metabolic profile of fatty acids in plasma and liver tissue

We evaluated plasma levels of thirty-two fatty acids at 24, 48, and 64 weeks (S7 Table). At 24 weeks of age mice fed with the CD-HFFC and CS-HFFC diet had significantly lower plasma levels of 7 fatty acids (of the 32 evaluated) compared to mice fed with the control diet. 85.7% of those fatty acids were polyunsaturated fatty acids (PUFAs). At 48 weeks of age, 7 of the 32 fatty acids had lower plasma levels in mice fed with the CD-HFFC and CS-HFFC diet compared to the control diet while 1 fatty acid (DHA) was lower in CD-HFFC fed mice only. 50% of fatty acids altered at 48 weeks were PUFAs. At 64 weeks of age, 12 fatty acids were significantly lower in the plasma of mice fed with the CD-HFFC and CS-HFFC mice, 1 was significantly higher in the plasma of mice fed with the CS-HFFC diet that developed HCC (Mead acid; MUFA), and 1 was significantly higher in the plasma of both CD-HFFC fed and CS-HFFC fed mice with HCC (DPA; PUFA, n-6). 71.4% of fatty acids that were altered at 64 weeks were PUFAs.

Fatty acid levels were also analyzed in tissue samples taken from the same mice at 64 weeks of age. Overall, there were three fatty acids that were significantly reduced in the dysplastic and HCC tissue of CD-HFFC and CS-HFFC diet mice compared to liver tissue from control mice (Heptadecanoic acid, SFA; Arachidic acid, SFA; and Hexacosanoic acid, SFA) (S1 Fig). Furthermore, Behenic acid (SFA) was observed to be significantly reduced in CS-HFFC fed mice with HCC compared to CS-HFFC fed mice with dysplastic nodules (S1 Fig).

Three plasma fatty acids were significantly lower in mice fed with the CD-HFFC and CS-HFFC diets compared to mice fed with the control diet at all three time points: γ -linolenic acid (PUFA, n-6), docosahexaenoic acid (PUFA, n-3), and docosapentaenoic acid (PUFA, n-3) (S2A Fig). The levels of all of these PUFAs were significantly higher at 48 weeks in CS-HFFC fed mice with HCC (S2B Fig) and remained elevated at 64 weeks for DHA but decreased at 64 weeks for γ -linolenic acid and DPA. The plasma levels of these fatty acids had no observed differences over time in the CD-HFFC fed mice with HCC and control mice. The tissue levels of these fatty acids did not differ significantly among the different groups (S3 Fig).

### Plasma cytokine profile

Of all plasma cytokines evaluated at 32 weeks of age, two were significantly higher in the CS-HFFC fed mice compared to the CD-HFFC fed mice (JE, p = 0.002 and TIMP-1, p = 0.0235) (S4 Fig). Some other cytokines (such as BLC, C5/C5a, Sicam-1, KC, M-CSF, SDF-1) also exhibited higher levels than the remaining cytokines, albeit, they were not different between the diet groups.

## Discussion

In this work we demonstrated the development of unhealthy diet (high fat/fructose/cholesterol) related NASH-HCC in two different contexts: lean NASH HCC in the setting of choline deficiency and obese NASH-HCC in the setting of choline supplementation. Mice fed with the CD-HFFC diet had lower HCC-free survival compared to mice fed with the CS-HFFC diet. Furthermore, lean NASH-HCC showed faster HCC progression with regards to tumor growth (HCC appearing as early as 32 weeks) compared to obese NASH-HCC. Lean NASH-HCCs were also larger in size compared to obese NASH-HCCs at the same timepoint. Thus, in males choline deficiency in the context of unhealthy diet results in accelerated hepatocarcinogenesis in the context of normal weight compared to choline supplementation in the context of obesity. It has been previously shown that choline supplementation in mice, albeit at higher levels and in a different mouse strain, results in attenuation of high fat diet-induced HCC [20].

Interestingly, insulin resistance and glucose intolerance were evident in obese CS-HFFC mice, with both male and female mice developing glucose intolerance by 48 weeks, male mice developing insulin resistance by 48 weeks and females transiently developing insulin resistance at 48 weeks. In contrast, only female CD-HFFC fed mice developed glucose intolerance after 24 weeks and only male mice transiently developed insulin resistance at around 48 weeks. Thus, persistent insulin resistance and glucose intolerance are features in the obese NASH-HCC model, whereas transient insulin resistance and glucose intolerance are features of lean NASH-HCC. This is consistent with human NASH, in which lean NASH patients appear to have a better metabolic profile compared to obese NASH patients [23].

With regards to plasma lipid profile, obese CS-HFFC fed mice (male and female) had higher levels of plasma cholesterol and triglycerides compared to lean CD-HFFC fed male mice. The latter may reflect potential retention of triglycerides in the liver of CD-HFFC fed mice [24]. While we did not assess plasma HDL, LDL, and VLDL levels specifically, previous reports show reduced plasma VLDL and unaffected plasma HDL levels in rats fed a choline deficient diet, suggesting that the higher total cholesterol levels we observed may be mostly due to HDL [25].

Both lean and obese mice fed with the CD-HFFC and CS-HFFC diets, respectively, experienced liver damage as evidenced by elevated plasma levels of ALT and AST. Lean mice fed with the CD-HFFC diet had higher levels of these enzymes at earlier time points suggesting more pronounced liver damage early on. At 20 weeks of age 100% of the obese CS-HFFC fed mice (male and female), 97.2% of male lean CD-HFFC fed mice, and 70% of lean CD-HFFC females had developed NASH. This implies differential effects of the diet by gender, with females experiencing lower prevalence of NASH in the context of choline deficiency. Obesity appears to have a bigger impact on the metabolic profile of females, worsening their metabolic profile that is characterized by glucose intolerance and insulin resistance. Thus, obesity plays a bigger role in NASH development in females whereas males develop NASH to the same extent in the context of normal weight and obesity. Such gender differences have been observed previously in mice [26]. In addition, lean females fed with the CD-HFFC only developed dysplastic nodules but not HCC at the endpoint, in contrast to obese females fed with the CS-HFFC diet who developed HCC at the endpoint. Lean and obese males developed HCC at the endpoint with similar penetrance. Therefore, obesity seems to provide the right context for tumor progression in females, whereas in males other mechanisms may be driving hepatocarcinogenesis irrespective of obesity. It is possible that in females, estrogen levels are protective of NASH development and consequently HCC in the context of normal weight [27]. However, in the context of obesity, estrogen signaling may be suppressed allowing for NASH and HCC progression.

Consistent with earlier liver damage in lean CD-HFFC fed mice compared to obese CS-HFFC mice, lean CD-HFFC-fed mice show more fibrosis and steatosis in their livers at 20 weeks compared to obese CS- HFFC-fed mice. This observation is reversed at the endpoint where obese CS-HFFC mice show more liver fibrosis and steatosis compared to CD-HFFC mice. Inflammation is lower at 20 weeks in obese CS-HFFC fed female mice compared to lean CD-HFFC fed female mice. The inflammation pattern is reversed at the endpoint with obese CS-HFFC fed mice showing higher levels of inflammation compared to lean CD-HFFC fed mice. It might be that in the context of choline deficiency and normal weight, changes in the liver microenvironment (inflammation and fibrosis) happen earlier and support initiation of hepatocarcinogenesis but may not be driving tumor progression. It could be that epigenetic changes drive tumor progression given the role of choline in one-carbon metabolism and supply of methyl groups for methylation [28]. In contrast, in the context of choline supplementation and obesity similar changes in the liver microenvironment occur later on and may be driving liver cancer initiation and progression. Interestingly, plasma levels of TIMP-1 and JE cytokines were higher in obese CS-HFFC further supporting the notion that the inflammatory microenvironment may be driving tumor progression in the context of obesity. Increased levels of TIMP-1 have been found to be associated with poor prognosis in gastric cancer, which relates to the role of matrix metalloproteinases in degrading the ECM [29]. A similar scenario may hold for obese NASH-HCC.

Given that NASH-HCC both in the context of obesity and normal weight is driven by an unhealthy diet, fatty acid metabolism is altered during disease progression. Interestingly, as observed in previous work [15], the plasma levels of a number of PUFAs were reduced in both settings consistent with their anti-carcinogenic role. Interestingly, PUFA levels remained low during the entire disease progression in lean mice, however, they increased and remained increased in obese mice, perhaps contributing to delayed tumor progression in the context of obesity. PUFAs have been suggested to have beneficial effects in improving metabolism in the context of obesity as well as improving insulin resistance [30]. It may be that increased levels of PUFAs may be counteracting the metabolic effects of obesity and given their anti-carcinogenic role may be contributing to delayed tumor progression. Thus, PUFA supplementation may have a beneficial effect in counteracting hepatocarcinogenesis in lean NASH-HCC. Furthermore, plasma mead acid levels were higher in CS-HFFC fed mice compared to CD-HFFC fed mice. Mead acid has been shown to suppress breast carcinogenesis and it is possible it may have a similar effect in liver tissue, thus contributing to delayed hepatocarcinogenesis in obese mice with NASH-HCC [31].

## Conclusions

An unhealthy diet high in trans-fat, cholesterol and fructose contributes to hepatocarcinogenesis both in the context of obesity and normal weight. Tumor progression is faster in males and slower in females in the context of normal weight compared to obesity, suggesting obesity has differential effects in carcinogenesis according to gender. The mouse models generated are great resources for studying lean and obese NASH-HCC and better understanding the mechanisms of hepatocarcinogenesis in these two contexts.

## Supporting information

S1 Table Control, CD-HFFC diet, and CS-HFFC diet consumption. Control, choline deficient, high trans-fat, fructose, and cholesterol (CD-HFFC), and choline supplemented, high trans-fat, fructose, and cholesterol (CS-HFFC) diet composition. Diets are custom mixed from Research Diets in New Brunswick, New Jersey. *Fat is mostly from palm oil.
(DOCX)

S2 Table The table shows the thirty-two fatty acids that were investigated. (DOCX)

S3 Table Coordinates for 40 cytokines tested in cytokine assay. (DOCX)

S4 Table Percent weight gain by diet type. Percent weight gain was calculated at 4 and 64 weeks of age to assess weight gain in the three groups of mice.
(DOCX)

S5 Table Gross observations of male mice fed with the control, CD-HFFC, and CS-HFFC diets upon necropsy. Mice fed with the CD-HFFC and CS-HFFC diets had larger livers, significantly higher liver to body weight ratios and enlarged spleens compared to mice fed with the control diet. * P-value for one-way ANOVA **P-value for t-test between CD-HFFC and CS-HFFC.
(DOCX)

S6 Table Gross observations of female mice fed with the control, CD-HFFC, and CS-HFFC diets upon necropsy. Mice fed with the CD-HFFC and CS-HFFC diets had larger livers, significantly higher liver to body weight ratios and enlarged spleens compared to mice fed with the control diet. * P-value for one-way ANOVA **P-value for t-test between CD-HFFC and CS-HFFC.
(DOCX)

S7 Table Fatty acids that were significantly different between mice fed with the control, CD-HFFC, and CS-HFFC diets. Bolded fatty acids are significant at all 3 time points.
(DOCX)

S8 Table Average steatosis (0–3), ballooning (0–2), and inflammation (0–3) for male and female mice at 20 and 64 weeks of age. None of the mice on the control diet had steatosis, ballooning, or inflammation at biopsy or endpoint. *T-test between CD-HFFC and CS-HFFC.
(DOCX)

S9 Table Steatosis, inflammation, and ballooning scores for mice at 20 and 64 weeks of age. (DOCX)

S1 Fig Fatty acid levels by diet at 64 weeks. Tissue fatty acid levels in male mice fed with the CD-HFFC (CD) diet with HCC and dysplastic (Dysp) nodules, mice fed with the CS-HFFC (CS) diet with HCC and dysplastic nodules, and mice fed with the control diet. ^ᵻ^ng/ug. ^a^ Significantly different than control. ^b^ CS Dysp and CS HCC are significantly different. (A-C) Levels of heptadecanoic, arachidic, and hexacosanoic acid in mice with dysplastic nodules and HCC fed both the CD and CS diet were significantly different than in control mice. (D) Levels of behenic acid were significantly different between mice fed the CS diet with dysplastic nodules and HCC.
(TIF)

S2 Fig Plasma fatty acid levels by diet type. (A) Plasma fatty acid levels in male mice fed with the CD-HFFC (CD) diet with HCC and dysplastic (Dysp) nodules, mice fed with the CS-HFFC (CS) diet with HCC and dysplastic nodules, and mice fed with the control diet. Mice with nodules (dysplastic and HCC) had lower plasma levels of specific fatty acids than control mice at 24, 48, and 64 weeks of age. (B) Trends of plasma fatty acid levels over course of study for each diet type. No concentration levels were observed in the control or CD-HFFC fed mice however CS-HFFC mice exhibited levels that increased at 48 weeks that decreased by 64 weeks of age.
(TIF)

S3 Fig Fatty acid levels by diet at 24, 48, and 64 weeks. Plasma and tissue fatty acid levels in male mice fed with the CD-HFFC (CD) diet with HCC and dysplastic (Dysp) nodules, mice fed with the CS-HFFC (CS) diet with HCC and dysplastic nodules, and mice fed with the control diet. ^ᵻ^ng/ug.
(TIF)

S4 Fig Plasma cytokine levels in male CD-HFFC and CS-HFFC fed mice at 32 weeks of age. (TIF)

S1 Dataset Minimal data set containing relevant data from study. (XLSX)

## Abbreviations

NAFLD: non-alcoholic fatty liver disease
NASH: non-alcoholic steatohepatitis
HCC: hepatocellular carcinoma
CS-HFFC: choline supplemented, high trans-fat, fructose, and cholesterol
CD-HFFC: choline deficient, high trans-fat, fructose, and cholesterol
