# Supplementary material for: Differential progression of unhealthy diet-induced hepatocellular carcinoma in obese and non-obese mice
Source: PLoS One. 2022 Aug 22;17(8):e0272623. doi: 10.1371/journal.pone.0272623 (PMC9394802; doi:10.1371/journal.pone.0272623)
Supplement: S1 Table — Control, choline deficient, high trans-fat, fructose, and cholesterol (CD-HFFC), and choline supplemented, high trans-fat, fructose, and cholesterol (CS-HFFC) diet composition. Diets are custom mixed from Research Diets in New Brunswick, New Jersey. *Fat is mostly from palm oil. (DOCX) [file pone.0272623.s001.docx]

|  | Control Diet | CD-HFFC Diet | CS-HFFC Diet |
| --- | --- | --- | --- |
| Diet Number | D16120211 | D17071001 | D18091706 |
| Fat (% kcal) | 12 | 44.9* | 44.9* |
| Protein (% kcal) | 20 | 20 | 20 |
| Carbohydrate (% kcal) | 70 | 35.1 | 35.1 |
| Fructose (% kcal) | 0 | 29.1 | 29.1 |
| Cholesterol (% g) | 0 | 2 | 2 |
| Choline (% g) | 0.2 | 0 | 0.2 |
| Total Calories | 4,057 | 4,057 | 4,057 |

Supplemental Table 1. Control, choline deficient, high trans-fat, fructose, and cholesterol ​(CD-HFFC), and choline supplemented, high trans-fat, fructose, and cholesterol (CS-HFFC) diet composition. ​ Diets are custom mixed from Research Diets in New Brunswick, New Jeresy. *Fat is mostly from palm oil
