# Supplementary material for: Differential progression of unhealthy diet-induced hepatocellular carcinoma in obese and non-obese mice
Source: PLoS One. 2022 Aug 22;17(8):e0272623. doi: 10.1371/journal.pone.0272623 (PMC9394802; doi:10.1371/journal.pone.0272623)
Supplement: S2 Table — (DOCX) [file pone.0272623.s002.docx]

| Fatty Acids​ | | |
| --- | --- | --- |
| Saturated fatty acids ​  (SFA)​ | Monounsaturated fatty acids ​  (MUFAs)​ | Polyunsaturated fatty acids ​  (PUFAs)​ |
| Lauric acid (C12)​  Myristic acid (C14)​  Pentadecanoicacid (C15)​  Palmitic acid (C16)​  Margaric acid (C17)​  Stearic acid (C18)​  Arachidicacid (C20)​  Behenic acid (C22)​  Lignocericacid (C24)​  Cerotic acid (C26)​ | Palmitoleic acid (16:1)​  Oleic acid (18:1)​  Eicosenoic acid (20:1)​  Mead acid (20:3)​  Heptadecenoicacid ​  Erucic acid (22:1)​  Nervonic acid (24:1)​ | Linoleic acid (ω-6) (18:2)​  Linolenic acids α(ω-3) & γ (ω-6) (18:3)​  Stearidonic acid (ω-3) (18:4)​  Eicosadienoic acid (ω-6) (20:2)​  Dihomo-γ-linolenicacid (ω-6) (20:3)​  Arachidonic acid (ω-6) (20:4)​  Eicosapentaenoicacid (ω-3) (20:5)​  Docosadienoic acid (ω-6) (22:2)​  Docosatrienoicacid (ω-3) (22:3)​  Adrenic acid (ω-6) (22:4)​  Clupanodonic acid (ω-3) (22:5)​  Osbond acid (ω-6) (22:5)​  Docosahexaenoic acid (ω-3) (22:6)​ |

Supplemental Table 2. The table shows the thirty-two fatty acids that were investigated.
