# Supplementary material for: Differential progression of unhealthy diet-induced hepatocellular carcinoma in obese and non-obese mice
Source: PLoS One. 2022 Aug 22;17(8):e0272623. doi: 10.1371/journal.pone.0272623 (PMC9394802; doi:10.1371/journal.pone.0272623)
Supplement: S3 Table — (DOCX) [file pone.0272623.s003.docx]

| Coordinate | Target | Coordinate | Target |
| --- | --- | --- | --- |
| A1, A2 | Positive Control | C17, C18 | IL-16 |
| A23, A24 | Positive Control | C19, C20 | IL-17 |
| B1, B2 | BLC | C21, C22 | IL-23 |
| B3, B4 | C5/C5a | C23, C24 | IL-27 |
| B5, B6 | G-CSF | D1, D2 | IP-10 |
| B7, B8 | GM-CSF | D3, D4 | I-TAC |
| B9, B10 | I-309 | D5, D6 | KC |
| B11, B12 | Eotaxin | D7, D8 | M-CSF |
| B13, B14 | Sicam-1 | D9, D10 | JE |
| B15, B16 | IFN-G | D11, D12 | MCP-5 |
| B17, B18 | IL-1A | D13, D14 | MIG |
| B19, B20 | IL-1B | D15, D16 | MIP-1A |
| B21, B22 | IL-1RA | D17, D18 | MIP-1B |
| B23, B24 | IL-2 | D19, D20 | MIP-2 |
| C1, C2 | IL-3 | D21, D22 | RANTES |
| C3, C4 | IL-4 | D23, 24 | SDF-1 |
| C5, C6 | IL-5 | E1, E2 | TARC |
| C7, C8 | IL-6 | E3, E4 | TIMP-1 |
| C9, C10 | IL-7 | E5, E6 | TNF-A |
| C11, C12 | IL-10 | E7, E8 | TREM-1 |
| C13, C14 | IL-13 | F1, F2 | Positive Control |
| C15, C16 | IL-12 p70 | F23, 24 | Negative Control |

Supplemental Table 3. Coordinates for 40 cytokines tested in cytokine assay.
