# Supplementary material for: Differential progression of unhealthy diet-induced hepatocellular carcinoma in obese and non-obese mice
Source: PLoS One. 2022 Aug 22;17(8):e0272623. doi: 10.1371/journal.pone.0272623 (PMC9394802; doi:10.1371/journal.pone.0272623)
Supplement: S4 Table — Percent weight gain was calculated at 4 and 64 weeks of age to assess weight gain in the three groups of mice. (DOCX) [file pone.0272623.s004.docx]

|  | | Average Weight at 4 weeks | Average Weight at 64 weeks | Percent Weight Gained |
| --- | --- | --- | --- | --- |
| Control Diet | Male | 13.18 | 35.62 | 171% |
|  | Female | 13.02 | 30.66 | 131% |
| CD-HFFC Diet | Male | 13.76 | 34.98 | 154% |
|  | Female | 12.67 | 32.61 | 154% |
| CS-HFFC Diet | Male | 13.84 | 58.09 | 319% |
|  | Female | 15.11 | 55.90 | 266% |

Supplemental Table 4. Percent weight gain was calculated at 4 and 64 weeks of age to assess weight gain in the three groups of mice.
