# Supplementary material for: Differential progression of unhealthy diet-induced hepatocellular carcinoma in obese and non-obese mice
Source: PLoS One. 2022 Aug 22;17(8):e0272623. doi: 10.1371/journal.pone.0272623 (PMC9394802; doi:10.1371/journal.pone.0272623)
Supplement: S5 Table — Mice fed with the CD-HFFC and CS-HFFC diets had larger livers, significantly higher liver to body weight ratios and enlarged spleens compared to mice fed with the control diet. * P-value for one-way ANOVA **P-value for t-test between CD-HFFC and CS-HFFC. (DOCX) [file pone.0272623.s005.docx]

|  | Control  Males​​ (n = 31) | CD  Males​​ (n = 29) | CS  Males (n = 30) | P-value  Males​​ |
| --- | --- | --- | --- | --- |
| Average liver weight​​ (g) | 1.50^a^  (+/- 0.21)​​ | 4.37^b^  (+/- 0.93) | 5.99^c^  (+/- 0.53) | 1.11 × 10^-16*^ |
| Average liver size​​ (mm^2^) | 650.78^a^  (+/- 127.24) ​​ | 956.04​​^b^  (+/- 163.71) ​​ | 1013.63^b^  (+/- 169.97) | 1.16 ×10^-^​​^14*^ |
| Average spleen weight​​ (g) | 0.11^​​a^  (+/- 0.05) ​​ | 0.26^​​b^  (+/- 0.09) ​​ | 0.28^b^  (+/- 0.05) | 1.11 ×10^-16*^ |
| Liver weight: ​​body weight​​ | 0.04^a^ | 0.13​​^b^ | 0.10^c^ | 1.11 ×10^-​​16*^ |
| Max nodule number​​ | 0​​ | 46​​ | 49 | ​​ |
| Min nodule number​​ | 0​​ | 1​​ | 1 | ​​ |
| Average nodule #​​ | 0​​ | 16.46 | 20.17 | 0.237** |
| Median nodule #​​ | 0​​ | 11.5 | 16 | ​​ |
| Max nodule size​​ (mm^2^) | 0​​ | 150.62 | 200.1 | 0.064** |
| Min nodule size​​ (mm^2^) | 0​​ | 3.38 | 1.87 | 0.165** |
| Average nodule size​​ (mm^2^) | 0​​ | 33.90 | 30.36 | ^​^​0.578** |
| Median​​ nodule size (mm^2^) | 0​​ | 17.74 | 8.48 | 0.060** |
| Dysplastic nodules​​ # | Max: 0​​  Average: ​ 0​ | Max: ​27​  Average: ​ 14.46​ | Max: ​24  Average: ​ ​13.8 | ​​0.792** |
| Dysplastic nodule size | Max: 0  Average: 0 | Max: 114.33  Average: 30.22 | Max: 67.3  Average: 31.85 | 0.791** |
| HCC​​ # | Max: 0​​  Average: ​ 0​ | Max: ​3  Average: ​ 0.85 | Max: 4  Average: ​ 0.77 | 0.751** |
| HCC size | Max: 0  Average: 0 | Max: 202.5  Average: 91.29 | Max: 36  Average: 11.25 | 0.003** |

Supplemental Table 5. Gross observations of male mice fed the control, CD-HFFC, and CS-HFFC diets upon necropsy. Mice fed the CD-HFFC and CS-HFFC diets had larger livers, significantly higher liver to body weight ratios and enlarged spleens compared to mice fed the control mice. ^*^ P-value for one-way ANOVA **P-value for t-test between CD-HFFC and CS-HFFC
