# Supplementary material for: Differential progression of unhealthy diet-induced hepatocellular carcinoma in obese and non-obese mice
Source: PLoS One. 2022 Aug 22;17(8):e0272623. doi: 10.1371/journal.pone.0272623 (PMC9394802; doi:10.1371/journal.pone.0272623)
Supplement: S6 Table — Mice fed with the CD-HFFC and CS-HFFC diets had larger livers, significantly higher liver to body weight ratios and enlarged spleens compared to mice fed with the control diet. * P-value for one-way ANOVA **P-value for t-test between CD-HFFC and CS-HFFC. (DOCX) [file pone.0272623.s006.docx]

|  | Control  Females​​  (n = 5) | CD-HFFC  Females​​  (n = 10) | CS-HFFC  Females​​  (n = 10) | P-value  Females​​ |
| --- | --- | --- | --- | --- |
| Average liver weight​​ (g) | 1.46^x^  (+/- 0.14)​​ | 3.83^y^  (+/- 0.38)​​ | 5.86^z^  (+/- 0.52) | 3.59 × 10^-14*^ |
| Average liver size​​ (mm^2^) | 661.8^x^  (+/- 129.73)​​ | 1040.2​​^y^  (+/- 106.94)​​ | 938^y^  (+/- 207.60) | 0.0019* |
| Average spleen weight​​ (g) | 0.12​​^x^  (+/- 0.04)​​ | 0.33^​​y^  (+/- 0.21)​​ | 0.28^y^  (+/- 0.06) | 0.035* |
| Liver weight :​​ body weight​​ | 0.05^x^ | 0.12^​​y^ | 0.10^y^ | 3.11 ×10^-​​10*^ |
| Max nodule number​​ | 0^​​^ | 63​​ | 49 | ​​ |
| Min nodule number​​ | 0​​ | 1​​ | 1 | ​​ |
| Average nodule #​​ | 0^​​^ | 27.3 | 27.5 | 0.977​** |
| Median nodule #​​ | 0^​​^ | 24.5 | 24 | ​​ |
| Max nodule size​​ (mm^2^) | 0^​​^ | 129.5 | 177.90 | 0.100** |
| Min nodule size​​ (mm^2^) | 0^​​^ | 8.20 | 1.90 | 0.325** |
| Average nodule size​​ (mm^2^) | 0^​​^ | 22.95 | 21.98 | 0.874** |
| Median​​ nodule size (mm^2^) | 0^​​^ | 12.70​​ | 9.15 | 0.576** |
| Dysplastic nodules​​ # | Max: 0  Average: 0​​ | Max: 50  Average:​ 26.2 | Max: 38  Average:​ ​24.6 | ​​0.769** |
| Dysplastic nodule size | Max: 0  Average: 0 | Max: 64  Average: 22.95 | Max: 37  Average: 22.08 | 0.887** |
| HCC​​ # | Max: ​0​  Average:​ 0​ | Max: 0  Average: 0 | Max: 1  Average:​ ​0.5 | 0.007** |
| HCC size | Max: 0  Average: 0 | Max: 0  Average: 0 | Max: 25  Average: 14.80 | 0.018** |

Supplemental Table 6. Gross observations of female mice fed the control, CD-HFFC, and CS-HFFC diets upon necropsy. Mice fed the CD-HFFC and CS-HFFC diets had larger livers, significantly higher liver to body weight ratios and enlarged spleens compared to mice fed the control mice. ^*^ P-value for one-way ANOVA **P-value for t-test between CD-HFFC and CS-HFFC
