# Supplementary material for: Differential progression of unhealthy diet-induced hepatocellular carcinoma in obese and non-obese mice
Source: PLoS One. 2022 Aug 22;17(8):e0272623. doi: 10.1371/journal.pone.0272623 (PMC9394802; doi:10.1371/journal.pone.0272623)
Supplement: S7 Table — Bolded fatty acids are significant at all 3 time points. (DOCX) [file pone.0272623.s007.docx]

| Plasma Fatty Acids at 24 weeks | P-value |  | Plasma Fatty Acids at 48 weeks | P-value |  | Plasma Fatty Acids at 64 weeks | P-value |
| --- | --- | --- | --- | --- | --- | --- | --- |
| **Heptadecanoic acid** | **0.0051** |  | Lauric Acid | 0.041 |  | **Heptadecanoic acid** | **0.00003** |
| Stearidonic acid | 0.0033 |  | Palmitoleic Acid | 0.011 |  | Stearidonic acid | 0.0016 |
| α-Linoleic acid | 0.0054 |  | **Heptadecenoic Acid** | **0.048** |  | **γ-Linoleic acid** | **0.0006** |
| **γ-Linoleic acid** | **0.0087** |  | **γ-Linoleic acid** | **0.014** |  | α-Linoleic acid | 0.002 |
| Eicosapentaenoic acid | 0.0033 |  | Linoleic acid | 0.039 |  | Eicosapentaenoic acid | 0.0006 |
| **Docosahexaenoic acid** | **0.0032** |  | Eicosenoic acid | 0.022 |  | Mead acid | 0.0075 |
| **Docosapentaenoic acid ω-3** | **0.0016** |  | **Docosahexaenoic acid** | **0.0043** |  | Eicosadienoic acid | 0.0175 |
|  |  |  | **Docosapentaenoic acid ω-3** | **0.01** |  | Eicosenoic acid | 0.041 |
|  |  |  |  |  |  | **Docosahexaenoic acid** | 0.0003 |
|  |  |  |  |  |  | **Docosapentaenoic acid ω-3** | **0.0004** |
|  |  |  |  |  |  | Docosapentaenoic acid ω-6 | 0.0179 |
|  |  |  |  |  |  | Docosatrieoic acid | 0.045 |
|  |  |  |  |  |  | Docosenoic acid | 0.015 |
|  |  |  |  |  |  | Nervonic acid | 0.021 |

Supplemental Table 7. Fatty acids that were significantly different between control, CD-HFFC, and CS-HFFC diets. Bolded fatty acids are significant at all 3 time points.
