# Supplementary material for: Differential progression of unhealthy diet-induced hepatocellular carcinoma in obese and non-obese mice
Source: PLoS One. 2022 Aug 22;17(8):e0272623. doi: 10.1371/journal.pone.0272623 (PMC9394802; doi:10.1371/journal.pone.0272623)
Supplement: S8 Table — None of the mice on the control diet had steatosis, ballooning, or inflammation at biopsy or endpoint. *T-test between CD-HFFC and CS-HFFC. (DOCX) [file pone.0272623.s008.docx]

|  | Male | | | Female | | |
| --- | --- | --- | --- | --- | --- | --- |
|  | CD-HFFC Diet | CS-HFFC Diet | P-value* | CD-HFFC Diet | CS-HFFC Diet | P-value* |
| Average Steatosis at 20 Weeks | 1.82 | 1.6 | 0.174 | 2.2 | 1.4 | 0.006 |
| Average Inflammation at 20 weeks | 1.28 | 1.23 | 0.211 | 1.4 | 1.2 | 0.006 |
| Average Ballooning at 20 weeks | 1.4 | 1.56 | 0.683 | 0.8 | 1.6 | 0.355 |
| Average Steatosis at 64 weeks | 1.57 | 2.46 | 3.81 × 10^-7^ | 1.5 | 2.2 | 0.004 |
| Average Inflammation at 64 weeks | 1.34 | 1.93 | 0.032 | 1.5 | 1.8 | 0.62 |
| Average Ballooning at 64 weeks | 1.34 | 1.63 | 0.002 | 1.7 | 1.8 | 0.38 |

Supplemental Table 8. Average steatosis (0-3), ballooning (0-2), and inflammation (0-3) for male and female mice at 20 and 64 weeks of age. None of the mice on the control diet had steatosis, ballooning, or inflammation at biopsy or endpoint. *T-test between CD-HFFC and CS-HFFC
