# Supplementary material for: Differential progression of unhealthy diet-induced hepatocellular carcinoma in obese and non-obese mice
Source: PLoS One. 2022 Aug 22;17(8):e0272623. doi: 10.1371/journal.pone.0272623 (PMC9394802; doi:10.1371/journal.pone.0272623)
Supplement: S9 Table — (DOCX) [file pone.0272623.s009.docx]

|  | Male | | | Female | | |
| --- | --- | --- | --- | --- | --- | --- |
|  | Control  Diet | CD-HFFC  Diet | CS-HFFC  Diet | Control  Diet | CD-HFFC  Diet | CS-HFFC  Diet |
| Steatosis at 20 weeks |  |  |  |  |  |  |
| 0 | 33  97% | 0 | 0 | 5  100% | 0 | 0 |
| 1 | 1  3% | 14  40% | 12  40% | 0 | 1  10% | 6  60% |
| 2 | 0 | 13  37% | 18  60% | 0 | 6  60% | 4  40% |
| 3 | 0 | 8  23% | 0 | 0 | 3  30% | 0 |
| Inflammation at 20 weeks |  |  |  |  |  |  |
| 0 | 32  94% | 0 | 0 | 4  80% | 0 | 0 |
| 1 | 1  3% | 25  71% | 23  77% | 1  20% | 6  60% | 8  80% |
| 2 | 1  3% | 10  29% | 7  23% | 0 | 4  40% | 2  20% |
| 3 | 0 | 0 | 0 | 0 | 0 | 0 |
| Ballooning at 20 weeks |  |  |  |  |  |  |
| 0 | 32  64% | 1  3% | 0 | 5  100% | 3  30% | 0 |
| 1 | 2  6% | 19  54% | 13  43% | 0 | 6  60% | 4  40% |
| 2 | 0 | 15  43% | 17  57% | 0 | 1  10% | 6  60% |
| Steatosis at 64 weeks |  |  |  |  |  |  |
| 0 | 32  100% | 0 | 0 | 5  100% | 0 | 0 |
| 1 | 0 | 12  46% | 1  4% | 0 | 5  50% | 0 |
| 2 | 0 | 13  50% | 14  46% | 0 | 5  50% | 8  80% |
| 3 | 0 | 1  4% | 15  50% | 0 | 0 | 2  20% |
| Inflammation at 64 weeks |  |  |  |  |  |  |
| 0 | 32  100% | 0 | 0 | 5  100% | 0 | 0 |
| 1 | 0 | 17  65% | 11  37% | 0 | 5  50% | 5  50% |
| 2 | 0 | 9  35% | 10  33% | 0 | 5  50% | 2  20% |
| 3 | 0 | 0 | 9  30% | 0 | 0 | 3  30% |
| Ballooning at 64 weeks |  |  |  |  |  |  |
| 0 | 32  100% | 0  65% | 0 | 5  100% | 0 | 0 |
| 1 | 0 | 17  35% | 11  37% | 0 | 3  30% | 2  20% |
| 2 | 0 | 9 | 19  63% | 0 | 7  70% | 8  80% |

Supplemental Table 9. Steatosis, inflammation, and ballooning scores for mice at 20 and 64 weeks of age.
