# Supplementary figures and images for: Differential progression of unhealthy diet-induced hepatocellular carcinoma in obese and non-obese mice
Source: PLoS One. 2022 Aug 22;17(8):e0272623. doi: 10.1371/journal.pone.0272623 (PMC9394802; doi:10.1371/journal.pone.0272623)

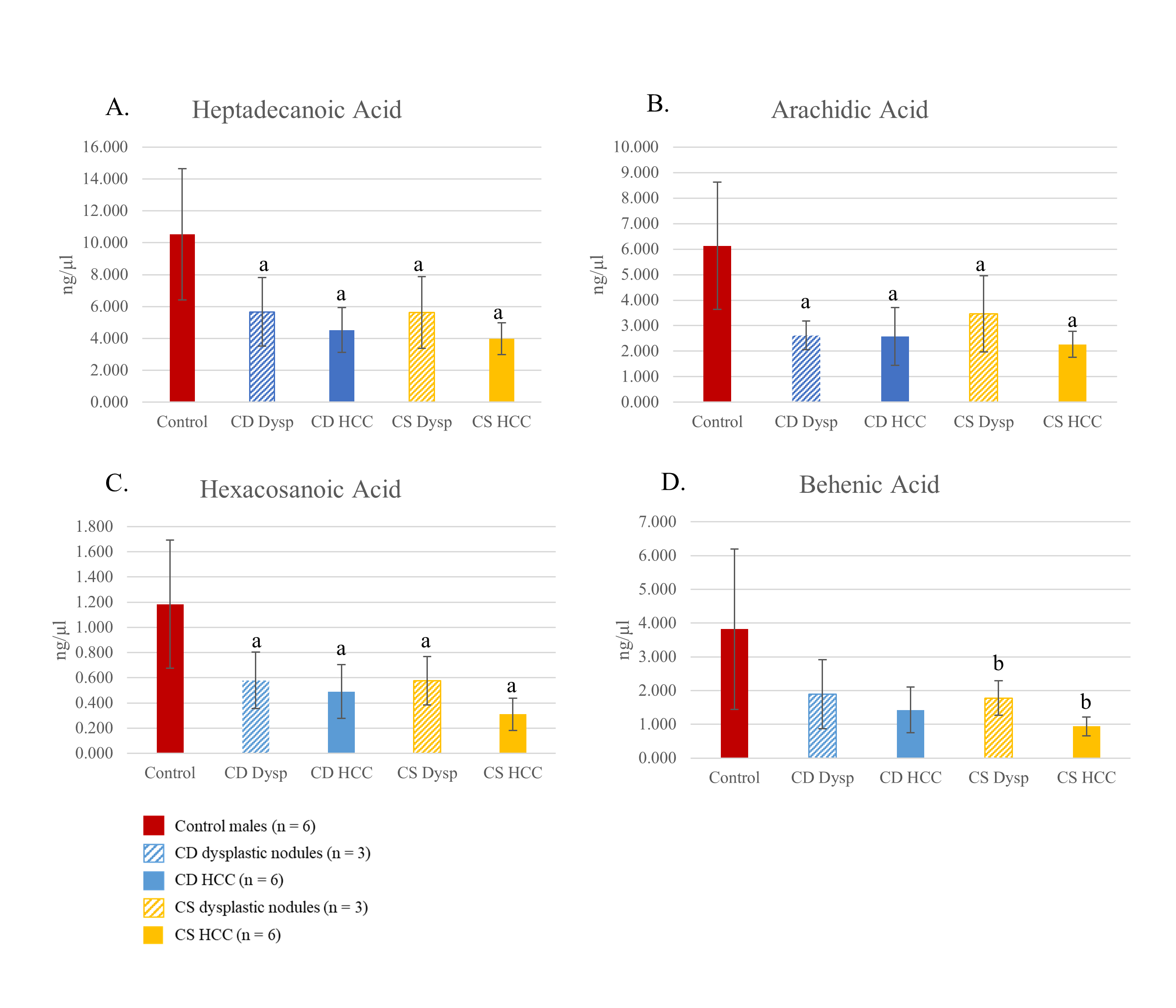

Supplement: S1 Fig — Tissue fatty acid levels in male mice fed with the CD-HFFC (CD) diet with HCC and dysplastic (Dysp) nodules, mice fed with the CS-HFFC (CS) diet with HCC and dysplastic nodules, and mice fed with the control diet. ᵻng/ug. a Significantly different than control. b CS Dysp and CS HCC are significantly different. (A-C) Levels of heptadecanoic, arachidic, and hexacosanoic acid in mice with dysplastic nodules and HCC fed both the CD and CS diet were significantly different than in control mice. (D) Levels of behenic acid were significantly different between mice fed the CS diet with dysplastic nodules and HCC. (TIF) [file pone.0272623.s010.tif]

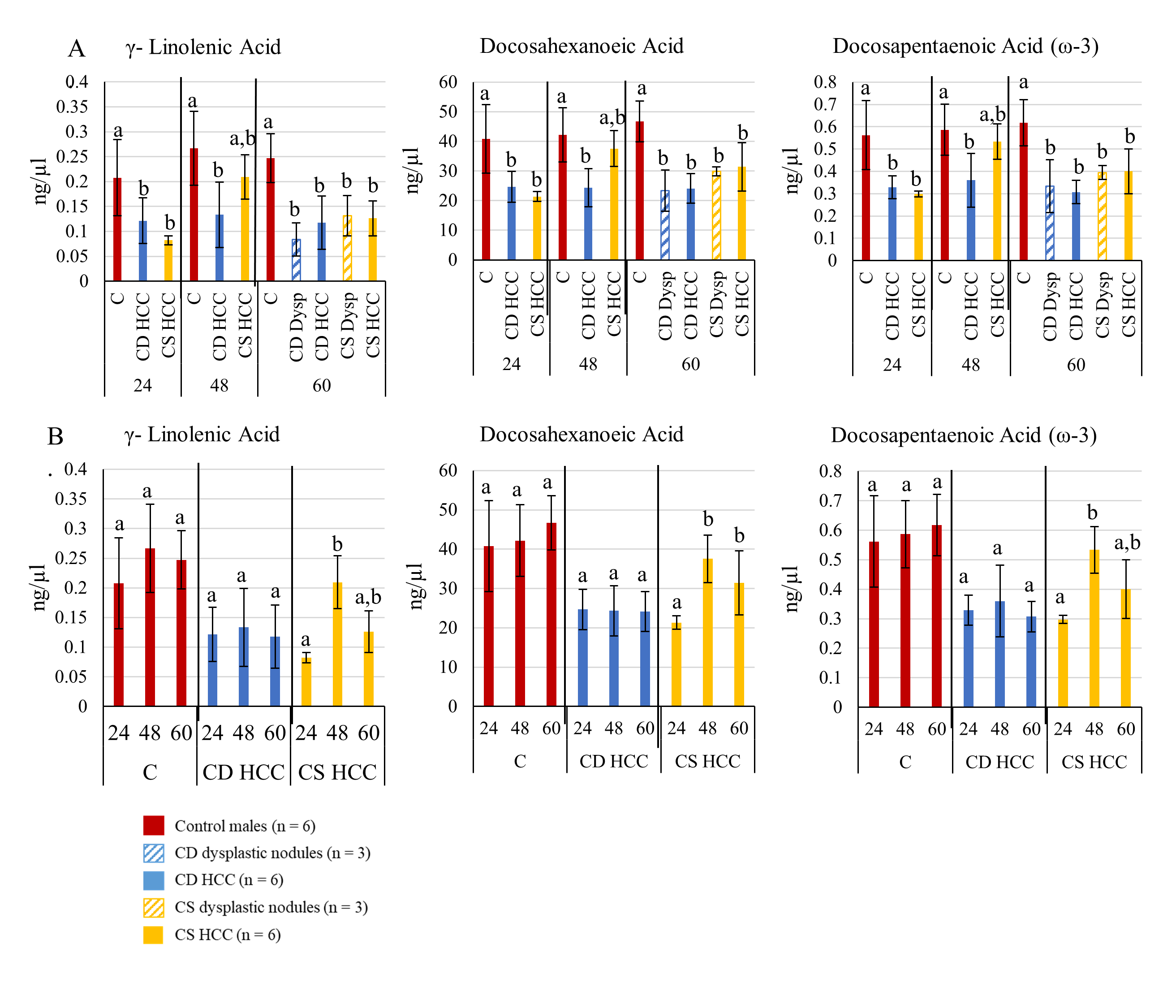

Supplement: S2 Fig — (A) Plasma fatty acid levels in male mice fed with the CD-HFFC (CD) diet with HCC and dysplastic (Dysp) nodules, mice fed with the CS-HFFC (CS) diet with HCC and dysplastic nodules, and mice fed with the control diet. Mice with nodules (dysplastic and HCC) had lower plasma levels of specific fatty acids than control mice at 24, 48, and 64 weeks of age. (B) Trends of plasma fatty acid levels over course of study for each diet type. No concentration levels were observed in the control or CD-HFFC fed mice however CS-HFFC mice exhibited levels that increased at 48 weeks that decreased by 64 weeks of age. (TIF) [file pone.0272623.s011.tif]

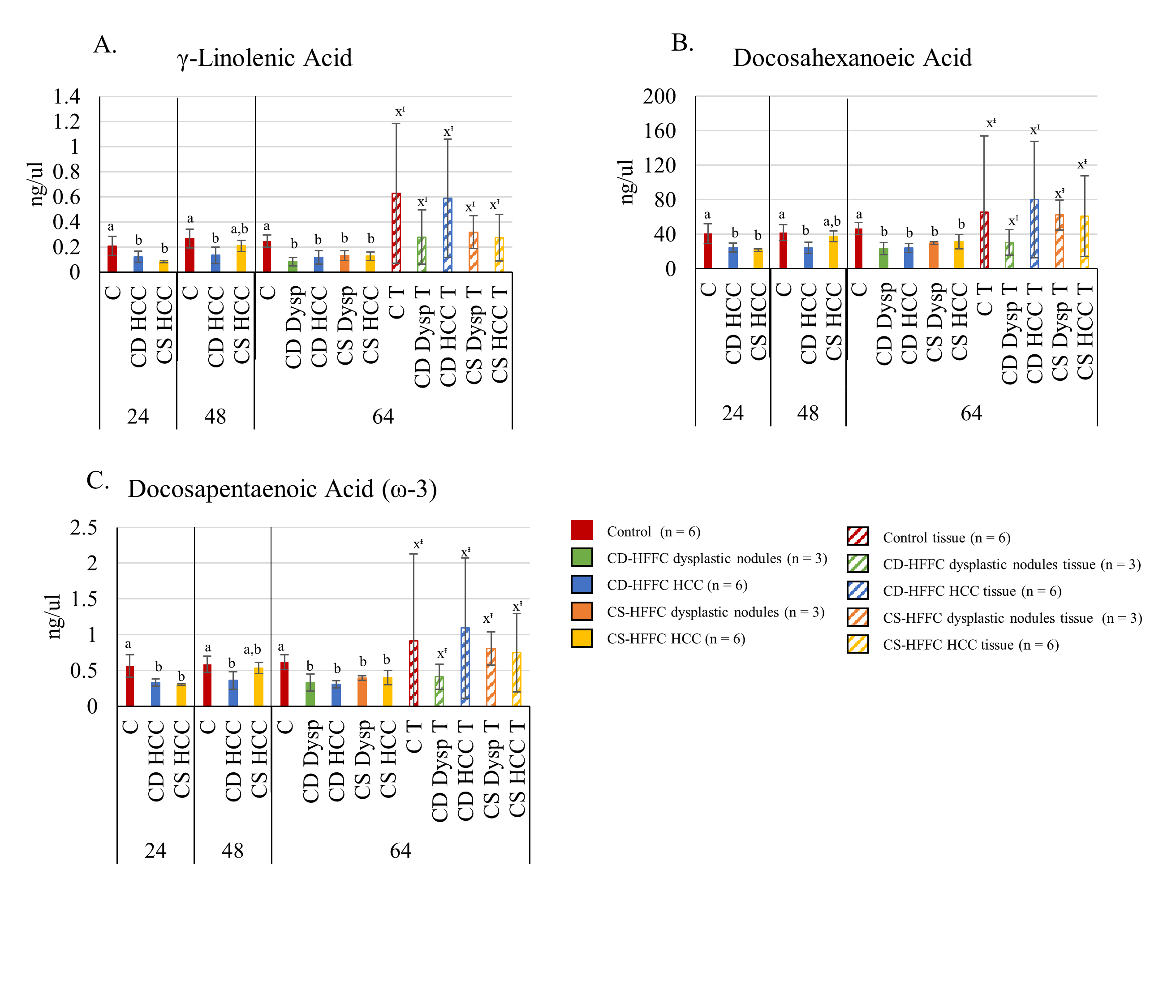

Supplement: S3 Fig — Plasma and tissue fatty acid levels in male mice fed with the CD-HFFC (CD) diet with HCC and dysplastic (Dysp) nodules, mice fed with the CS-HFFC (CS) diet with HCC and dysplastic nodules, and mice fed with the control diet. ᵻng/ug. (TIF) [file pone.0272623.s012.tif]

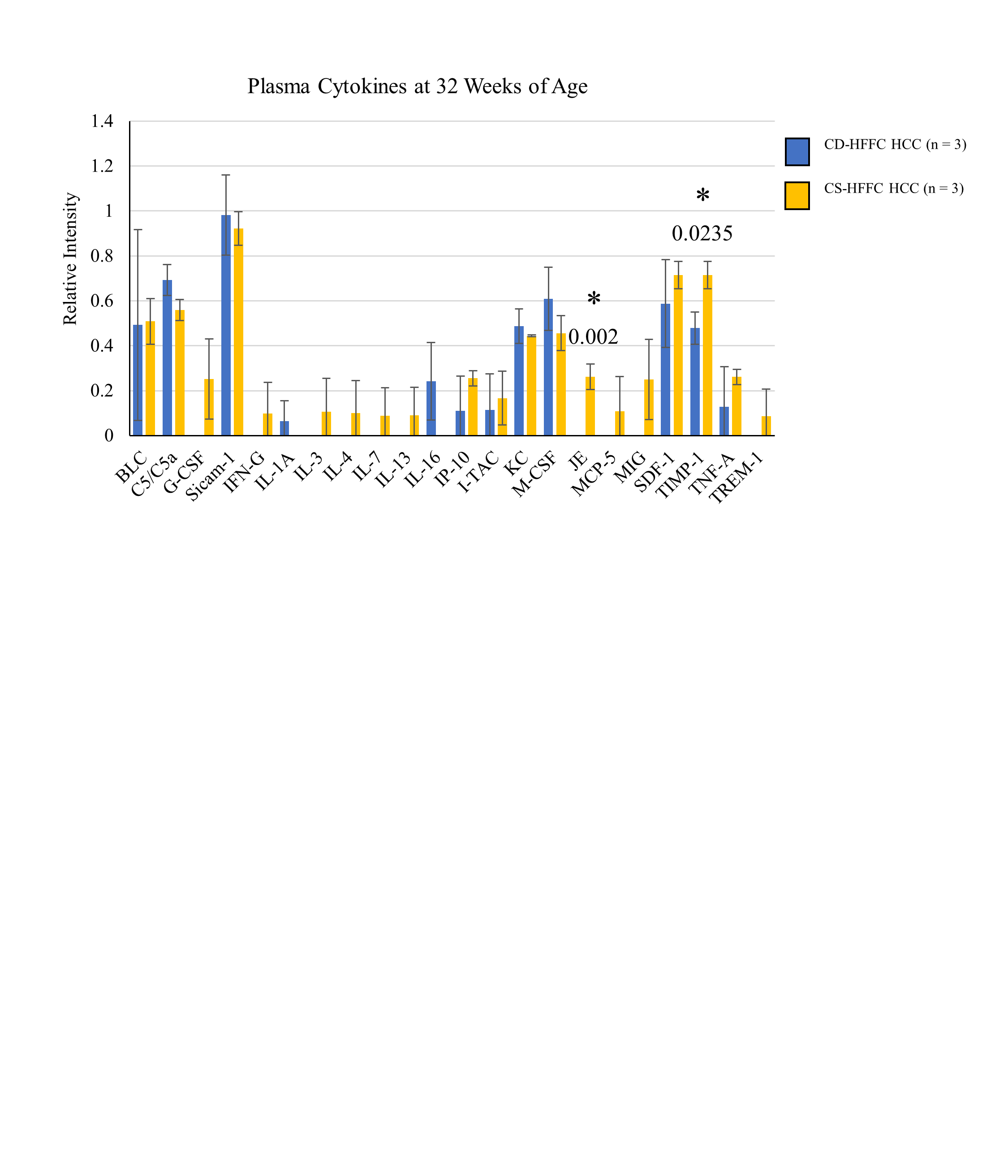

Supplement: S4 Fig — (TIF) [file pone.0272623.s013.tif]
